# Ternary spatial reconstruction of ecological migration in Jiaochang Village

**DOI:** 10.1016/j.heliyon.2024.e33720

**Published:** 2024-06-26

**Authors:** Xueyue Bai, Qihui Liu, Jingtian Ge, Cong Dong

**Affiliations:** a School of Landscape Architecture, Beijing Forestry University, 35 Qinghua East Road, Haidian District, Beijing, 100083, China; b College of Humanities and Development Studies, China Agricultural University, No. 2 Yuanmingyuan West Road, Haidian District, Beijing, 100193, China

**Keywords:** Ecological migration, Spatial reconstruction, Ternary space

## Abstract

In 2020, the total number of ecological migrants in China reached 48.73 million; To address this situation, this study focuses on Jiaochang Village in Sichuan Province as an example in order to examine the living spaces of ecological migrants after immigration. Based on Lefebvre's Ternary Space Theory, this study analyzed the spatial reconstruction of an ecological immigrant village on the basis of rural revitalization on three levels, i.e., residential space, social space, and spiritual space, by using the methods of geographic information comparison, field building surveys, structured interviews, and social network analysis. At the same time, by comparing the eco-migrants’ situations before and after relocation, in addition to comparing them with those of the indigenous villagers, the adaptation of the ecological migrants in the above-mentioned ternary space was examined. The results showed a difference in living space for the ecological immigrants and the indigenous people, but the difference in living space for the migrants before and after relocation was larger. There was little difference in social spaces for the migrants and the indigenous villagers. The unemployment rate of the ecological immigrants was higher, and the interaction intensity in their work network was smaller. The spiritual space of the immigrants appeared very different from that of the indigenous people. The migrants' spiritual condition was slightly worse, but the preservation of traditions was stronger for the immigrants than for the indigenous people. The living and social spaces of the ecological immigrants were basically reconstructed, whereas the spiritual space requires more time to be organized.

## 1 Background

In recent years, the Chinese government has committed to using ecological migration to improve the living conditions of residents in ecologically sensitive areas, reduce environmental pressure, and achieve ecological restoration and regeneration. In 2020, the total number of ecological migrants in China reached 48.73 million. However, ecological migration is a complex process involving many factors, including environmental, social, and economic issues, and although positive changes have been achieved in some cases, some challenges remain.

Ecological migration is defined as the movement of a population from its original place of residence to another area due to the deterioration of their original ecological environment or in order to improve and protect the ecological environment [1]. In the last few decades, with the increasing prominence of environmental problems and the increasing awareness of the negative impact of human activities on ecosystems, people have begun to pay attention to how to protect and restore the ecological environment. Ecological migration has been proposed and studied as a solution.

At first, research on ecological migration mainly focused on social adaptation, migrant rights and interests, protection policies, and cultural continuity for ecological migrants. Morvaridi (2004) studied the issue of resettlement and development rights in the Ilisu Dam project in Turkey [2], and Colchester (2004) analyzed the impact of protective policies on the rights and livelihoods of indigenous peoples [3]. Adams and Hutton (2007) explored the impact of protected areas on local communities [4], and Agrawal and Redford (2009) outlined the relationship between conservation actions and the displacement of local communities [5]. The study of Curran et al. (2009) focused on the impact of protected areas on the rural poor in Central Africa [6]. Ptackova (2011) studied the settlement of herdsmen in Tibet, China, and emphasized the importance of respecting the local culture and traditional lifestyle of ecological migrants [7]. Foggin (2011) proposed suggestions for improving ecological migration policies to ensure the protection of the environment while preserving the culture of local communities [8].

In recent years, research on ecological migration has mainly focused on environmental rights, traditional ecological knowledge, vulnerability assessment, heritage protection, and migration behavior and impact. Ma et al. (2019) studied the changes in the traditional ecological knowledge of forage plants in migrant villages in Ningxia, China [9]. Han et al. (2020) assessed the vulnerability of the human–environment system in the Liupanshan region of Ningxia, China, in order to promote the sustainable development of this region and provide an important reference for the subsequent expansion of ecological migration [10]. Shpakovskiy et al. (2020,2021) studied the issue of modern ecological migration through a legal analysis and formulated relevant suggestions [11,12]. Yang et al. (2020) studied the resettlement intentions of ecological migrants [13]. Dluhopolskyi (2019) conducted a legal analysis of the practical reasons for Ukrainian immigration [14]. Fang et al. (2020) analyzed the behavioral problems of immigrant adolescents in relation to the situation of adolescents in both the destination and the area of origin [15]. Xu (2021) evaluated the types and comprehensive effects of ecological migration in China [16]. However, the spatial reconstruction of ecological migration has not been thoroughly studied, and only some studies on this topic have been published. Zhang Tian et al. (2017) discussed the evolution and reconstruction of rural space from the perspective of resilience [17]. Chengjing Nie et al. (2020) evaluated spatial reconstruction with respect to the promotion of countryside tourism, providing references for its development [18].

Research on ecological migration has evolved from focusing on social adaptation and rights protection to encompassing more dimensions such as environmental rights, heritage protection, and ecological knowledge, indicating that existing studies have shifted from a singular focus to a multi-dimensional perspective on ecological migration issues. Currently, there is a lack of a comprehensive framework to understand and assess the multi-dimensional effects and long-term impacts of spatial reconstruction caused by ecological migration. The necessity for multi-dimensional research stems from the complexity of ecological migration, which involves social, environmental, and cultural aspects. Therefore, research needs to be holistic to fully understand its impacts and consequences. Spatial reconstruction is a very important aspect of the ecological migration process, directly affecting the quality of life and sustainable development of communities post-migration. The evolution from focusing solely on social adaptation and rights protection to including environmental rights, heritage protection, and ecological knowledge emphasizes the material aspects. These new research focuses have shifted attention more towards the material aspects, but the introduction of a spatial perspective further emphasizes the importance of 'people’ in the ecosystem. The interactions between ecological, social, and cultural factors are more complex in rural areas, thus the impact of ecological migration on these regions is more profound.

China has a large population and covers a wide geographical area; therefore, it faces great challenges in terms of ecological migration. Ecological migration in China mainly involves large-scale migration in the Three Gorges Reservoir area and the Qinghai–Tibet Plateau. The earliest ecological migration study regarded the ecological migration in the Three Gorges Reservoir area of Sichuan Province [19]. Then, Long (2014) studied the issue of land consolidation in rural China and emphasized its importance in promoting rural spatial reconstruction [20]. Chengju (2016) studied resettlement and livelihood reconstruction projects and revealed their potential of alleviating migrants’ poverty [21]. Zhao X. (2018) studied land-related issues associated with outward migration [22].

The Wenchuan earthquake was a serious earthquake disaster in Chinese history that resulted in a large number of casualties and extensive damage to buildings [23]. Many scholars have studied the situation in the affected areas after the Wenchuan earthquake [24,25]. Post-earthquake development and reconstruction have also been monitored [26]. In order to restore the ecological environment and protect people's lives, the Chinese government has adopted ecological immigration measures. Some mountainous areas and high-risk areas in the Wenchuan earthquake disaster area were identified as unsuitable for human habitation, and the government began to move residents from high-risk areas to safer places [27]. This study focuses on Mashan Village in Xuecheng Town, Sichuan Province, China, as an example of an ecological immigrant village after the 2008 Wenchuan earthquake. Using Lefebvre's tripartite spatial theory, this study analyzed the spatial reconstruction of an ecological migrant village after immigration by using the methods of geographic information comparison, field building surveys, structured interviews, and social network analysis. Identifying and discussing the problems faced by ecological immigrants living in different places can provide guidance for the construction of other ecological immigrant villages.

Jiaochang Village is located in the center of Xuecheng Town, Lixian County, Sichuan Province, China (103°13′ E, 31°33′ N), 23 km northeast of Lixian City, and 160 km away from the provincial capital Chengdu, at an average altitude of 1647 m in the mountainous arid valley climate zone. The Qiang, Tibetan, and Han ethnic groups (mainly Qiang) live together, as this area is an important node of the Tibetan and Qiang cultural corridor in western Sichuan. Jiaochang Village is located in the center of Xue Town, with an old school on the east side of the village. In the past, half of the Jiaochang villagers were moved into Mashan Village, 2.5 km in the northwest, and the original Mashan villagers were called the Mashan Village Group. In 2020, Mashan Village was merged into Jiaochang Village, and the combined settlement is now known as Jiaochang Village. Jiaochang Village belongs to Xuecheng Town, which was an important border and commercial town in the past. The ethnic composition of Jiaochang Village is complex, and it includes Han, Tibetan, and Qiang villagers, while the original Mashan villagers are mostly Qiang.

## 2 Research methods

### 2.1 Research framework

The core of Lefebvre's theory of space production is the sociality of space. Space is the product of society and is regarded as a kind of “social space”; it is not only a physical form but also a complex entity embedded in politics, economy, society and culture. He argues that every society and every mode of production has its own unique space [28]. Ecological immigration villages are one of the special phenomena appearing in the process of socialist modernization in China. The spatial production of the ecological migrant village can be understood with this theory. The village is the product of the subject's continuous practice and creation, from the social space that existed before the subject to the existing social space. Through the “displacement” of space, and changes to the original structure and order, a new order is created, that is, a new space. Spatial production is not only a material phenomenon but also a cultural phenomenon. The construction of new spaces in ecological migrant villages is the product of the interaction between social and economic development and production processes and is the core of the game among stakeholders with multiple interests, such as the government, village committees and villagers. In the theory of spatial production, Lefebvre put forwards “tripartite dialectics”, which mainly includes spatial practice, spatial representation and representational space.

Spatial practice is also known as perceived space. Social members produce and reproduce space through perception and action, thus forming living conventions, behavioural consensus and social structure. Spatial representation, also known as conceptualized space, describes the distribution of power and knowledge by the government, designers, etc., and presents the dominant social order, which is interrelated with production relations and its associated order. The representational space, also called the living space, is the real space of the social individual's life. Representational space bears the traces of spatial planning, and at the same time, it is hidden within the form of physical space, so it can truly reflect the face of social space. Spatial practice connects spatial representation with representational space, while spatial practice constructs representational space under the guidance and constraints of spatial representation. In the current research, some scholars have used triadic dialectics to analyze the production behaviours of ecological migrants, which can help us understand the interaction between ecological migrants and local society, including resource utilization, social structure and culture [29,30]. On the basis of previous studies, this study establishes a research framework based on ternary space theory, which is used to discuss the spatial reconstruction of ecological migration.

The framework divides the spatial reconstruction of ecological migration into three levels, namely, living space, social space and spiritual space, and analyses the spatial characteristics at each level. At the living space level, we pay attention to the spatial organization and layout of ecological migrants' residences. This includes the process by which they select and adapt to a new place of residence, as well as the physical environment and community characteristics of the new place of residence. At the social spatial level, we pay attention to the spatial dimension involved in the social practices of ecological migration. This includes their interaction with social structures and relationships, as well as their economic situation, and access to and consumption of public services. At the spiritual spatial level, we focus on the cultural spatial representation of ecological migration. This includes the spatial expression of their values, belief systems and cultural identities. By dividing the spatial reconstruction of ecological migration into three levels according to spatial practice, spatial representation and representation space and analysing the spatial characteristics at each level, this research framework can provide a comprehensive and in-depth perspective to help us better understand the spatial reconstruction of ecological migration and related social, cultural and environmental factors.

At the level of living space, we compared the regional characteristics of Jiaochang Village before and after the ecological immigration and then compared the living conditions and living satisfaction of the ecological immigrants and indigenous people. Jinhai et al. suggested that immigrants’ satisfaction with their new housing after relocation is mainly influenced by two factors. The first one is a good knowledge of the relocation policies, which will help them settle and reorganize; the second one concerns the housing space, as a large house will allow a high quality of life [31]. Fang and others argued that the more bedrooms there are in a house, the more content residents will likely feel [32]. In the analysis of the living space, the living situation of the ecological immigrants was examined by considering homestead areas, building areas, courtyard areas, and the number of rooms. To determine housing satisfaction, we considered the degree of satisfaction with housing, homestead size, and the environment.

At the level of social space, we compared the financial status, working status, and use of public social services by the ecological immigrants and the indigenous people in the village. Over the last few decades, the workplace became a fundamental part of Chinese cities [33]. Good economic conditions, meaningful work, and satisfactory family conditions can reflect the social conditions of farmers [34]. Public services are an important manifestation of social equity [35]. The use of public services by ecological migrants and indigenous people in Jiaochang Village can also reflect their differences in the reconstruction of social spaces. A comparison of the financial situation between migrants and indigenous residents was carried out by considering the specific average annual wages and savings. Their job situations were compared by examining the unemployment rates and performing a job network analysis. The use of public social services was compared by analyzing the frequency of visits to medical institutions, banks, and supermarkets by the two groups of residents.

At the level of the spiritual space, we compared the happiness and the preservation of traditions of the ecological immigrants and indigenous people in Jiaochang Village. The research of Rego, A. showed that a sense of belonging has a positive impact on people's degree of happiness [36]. Zhu, D.M. suggested that relocated residents would develop attachment to a place irrespective of the type of relocation method used [37]. The assimilation or protection of immigrant culture has always been one of the most studied issues in immigration policy [38]. Most scholars who study Qiang culture believe that Qiang culture needs to be preserved [39,40]. Happiness [41] and the preservation of cultural practices [42] are important components of the spiritual space that are not easy to quantify. Therefore, they were evaluated and compared in the two groups by assigning them a degree.

**Fig. 1:**
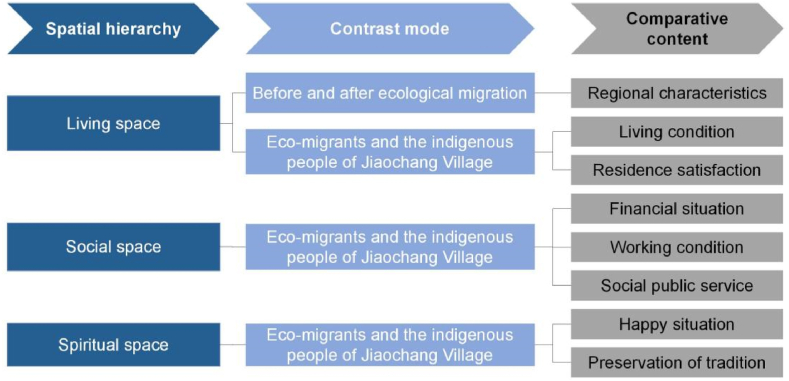
Research framework.

The three levels of space represent the establishment of progressive relations. The first to be defined after immigration is the living space, followed by the social space and, finally, the spiritual space. A diagram of our research framework is shown in Fig. 1.

### 2.2 Data source

The residential space was analyzed by using geographic information, building field surveys, and conducting structured interviews. The elevation data were obtained from a global elevation dataset developed by the United States Geological Survey (USGS). In addition, we referred to the satellite map on Google Earth and, finally, determined the elevation of the original village and that of the village after the migration after a field investigation.

Structured interviews and social network analysis were used to analyze the social space. Structured interviews were used for a comparison of spiritual spaces. The structured interviews were conducted in July and August 2021 and in January and February 2022 when the interviewees had finished working. In the first survey, households were selected by using the method of spatial sampling. Using a map of Jiaochang Village, the households were numbered, and then a random number table was generated. The first two surveys were conducted according to the sequence of the random number table. Each investigation was conducted by 3 teams, with each team comprising 2–3 people, and 1 team was able to speak the local dialect. The teams included interviewers and observers. Each interview lasted about 1 h. Finally, we collected the data and compiled a database.

### 2.3 Data analysis

First, all data were normalized, and data with different units or different orders of magnitude were converted into relatively uniform indicators for comparison and analysis. We used linear normalization. After data cleaning, missing value processing, and outlier processing, the data were attributed values from 0 to 1 or other specific ranges.

The linear scaling normalization (Min–Max normalization) formula is as follows:

$$X^{\prime }=\frac{\left(X-X_{\min}\right)}{\left(X_{\max}-X_{\min}\right)}$$

where X′ is the normalized value, X is the original value, X_min_ is the minimum value of the dataset, and X_max_ is the maximum value of the dataset. With this formula, the raw data were linearly organized within the range of [0, 1] or some other specified range.

After eliminating the dimensional differences in the data, the SPSS tool was used to conduct a *t*-test on the three levels of space for ecological migrants and indigenous people in order to determine whether the samples' means for the three levels of space were significantly different. The *t*-test was based on a key statistic, the *t*-value, which represented the size of the difference between two sample means relative to their standard error. By comparing the t-value with the critical value, it is possible to determine whether there was a significant difference between two samples' means. If the *t*-value was greater than the critical value, the null hypothesis could be rejected, that is, a significant difference existed between the two sample's means.

## 3 Results

### 3.1 Spatial practice of ecological migrant villages:Living space reconstruction

Living space plays a crucial role in the resettlement of ecological migrant villages, which is manifested in the change in the geographical location and spatial size of the village house base; this is the process and result of the specific stage of the construction of ecological migrant villages. At the same time, spatial practice has also become the main carrier of the social integration of ecological migrants and indigenous people. However, due to the differences in spatial practice, there are certain differences in the acquisition of homesteads between ecological migrants and indigenous people.

**Fig. 2:**
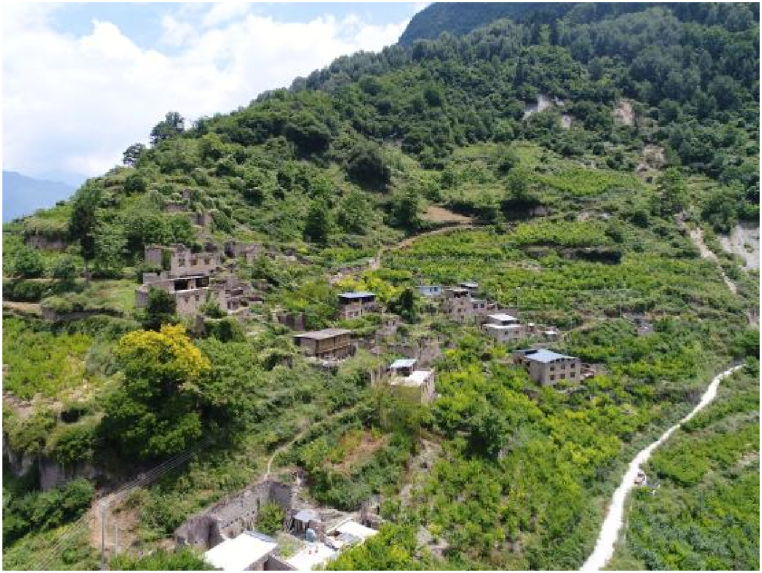
Former Mashan village.

**Fig. 3:**
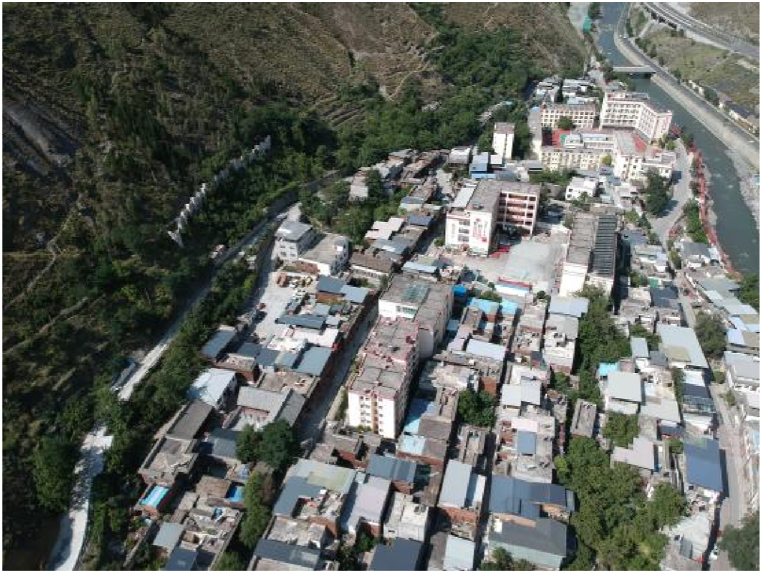
Current Jiaochang village.

The geographical features of the villages changed greatly after the relocation of the ecological migrants. Before the migrant relocation, the elevation of Jiaochangcun Village was 1594–1625 m, and that of Yuan Mashan Village was 2317–2395 m, with an elevation difference of about 700 m and an average temperature difference of about 4.2°. The two original villages also had different topographies and landforms, with Jiaochang Village being located in a high mountain valley zone and Mashan Village being among high mountains. In addition, Jiaochang Village developed on a small slope, while Mashan Village was spread on a large slope that faced northward. The original site of Mashan Village and that of the new Jiaochang Village are shown in Fig. 2, Fig. 3. At present, most of the houses in the original site of Mashan Village have been abandoned, and only a few houses are still in use. Fig. 2 shows an aerial photo of the former Mashan Village, and Fig. 3 shows the current Jiaochang Village.

The former Mashan villagers belong to the Qiang ethnic group, which mainly lives in the mountainous areas of western Sichuan Province. Most of the sites inhabited by Qiang people are located high on mountains [43], about 2000 m above sea level. This area is called the Qiang Village in the clouds. Jiaochang Village is located in the valley area at the foot of the mountains and is a multi-ethnic settlement area [44]. Because of the size of the site, the houses in Jiaochang Village are much smaller than those in Mashan Village, and the density of the houses is also higher, with no open space between the houses to grow crops. The living space of the villagers in Mashan Village changed greatly before and after the migrant relocation.

The living spaces of the indigenous people and ecological immigrants in Jiaochang Village were compared, as shown in Table 1. In terms of homestead areas, building areas, courtyard areas, number of rooms, number of bedrooms, and number of toilets, the average values for the indigenous villagers were found to be higher than those for the ecological immigrants. These data indicate that the indigenous people have more land and more spacious houses within the village.

**Table 1:** Comparison of the living conditions between ecological migrants and indigenous peoples.

	Mean Value for Ecological Migrants	Average Value for Indigenous People in Jiaochang Village
Homestead area	138.3333	167
Floor area	150.95	178.8667
Courtyard area	10.8947	42.1333
Number of rooms	7.8	8.7333
Number of bedrooms	4.3	5.4667
Number of toilets	1.55	2.3333

**Fig. 4:**
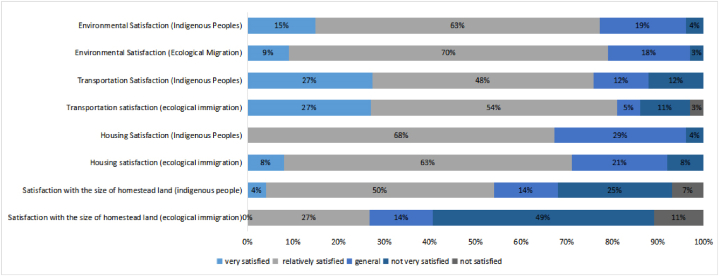
Comparison of living satisfaction between ecological immigrants and indigenous people.

The housing satisfaction of the indigenous people of Jiaochang Village and the ecological immigrants was compared, as shown in Fig. 4. The ecological immigrants and indigenous people were relatively satisfied with their housing, but the indigenous people appeared to be slightly more satisfied. Compared with that of the ecological immigrants, the satisfaction of the indigenous people regarding the size of their homestead appeared higher on the whole. The ecological migrants and indigenous people reported relatively high levels of satisfaction with transportation and the environment, with little difference overall. In summary, the indigenous people generally showed a higher degree of satisfaction with respect to the size of their homestead, while a small difference was found when comparing the satisfaction of ecological immigrants and indigenous people with housing, transportation, and environment. After relocation, the ecological immigrants had to settle in the living space of the indigenous people and adapt to smaller homestead areas and to houses with a lower number of rooms than in their original houses. Therefore, the ecological immigrants appeared slightly less satisfied with the size of their homestead than the indigenous villagers. However, regarding other aspects, the ecomigrants and the indigenous people appeared to be similarly satisfied.

### 3.2 Spatial representation of ecological immigrant village: the reconstruction of social space

The construction of social space plays an important role in the “relocation task” of the local government, and spatial representation is the means by which the local government conceptualizes this task. Local governments take the “relocation task” as a specific action goal. In the process of completing the relocation, the construction and allocation of social space become an important representation of the public functions by local governments. By improving and developing social space, local governments provide necessary public services to meet the basic needs of residents and improve their quality of life.

We compared the social space of the indigenous people and ecological immigrants in Jiaochang Village. In terms of average annual salary and savings, the average salary of the ecological immigrants was RMB 32,131.58, and the average salary of the indigenous people was RMB 50,747.83. The average salary of the indigenous people was significantly higher than that of the ecological immigrants. The average household savings of the ecomigrants was RMB 19,166.67, while the average household savings of the indigenous people was RMB 53,142.86, which was significantly higher than that of the ecomigrants.

**Fig. 5:**
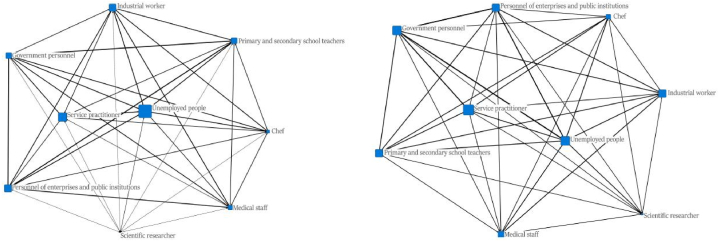
Ecological migrants' work network and indigenous villagers' work network.

In terms of the work situation, the unemployment rate of the ecological immigrants was 0.2, while that of the indigenous people was 0.1379, which was slightly lower than that of the ecomigrants. Ecological migrants' work network and indigenous villagers' work network diagram analysis is shown in Fig. 5. In the figure, the size of the nodes represents the proportion of individuals engaged in each type of work relative to the total population, while the thickness of the lines indicates the strength of connections between individuals across different types of work. We found that the work network of the ecological migrants was similar to that of the indigenous villagers, and the intensity of interactions between the nodes in the ecological migrants’ network was lower. The proportions of indigenous people in different occupations were more similar than for the ecological immigrants, who mainly worked in service industries or were unemployed.

**Fig. 6:**
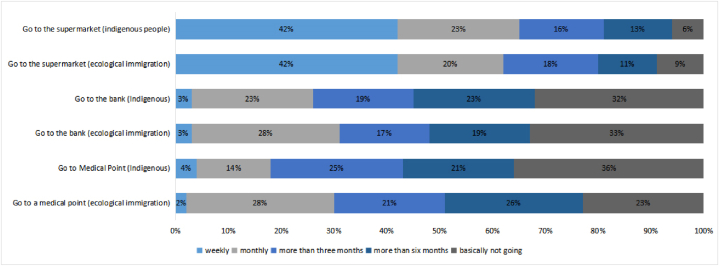
Comparison of the use of public social services between ecological migrants and indigenous villagers.

We then considered the villagers’ visits to relatives and friends during the Spring Festival. We found that the average number of ecological immigrants visiting family and friends for the Spring Festival was 7.96, while that of indigenous people was 4.68. Therefore, the average number of ecological immigrants traveling during the Spring Festival was higher.

In summary, the indigenous people showed higher average wages and household savings, while the ecological migrants traveled more during the Chinese New Year. In addition, the unemployment rate of the indigenous people was slightly lower than that of the ecomigrants.

We then analyzed the social space of the indigenous people and the ecological immigrants in Jiaochang Village. The use of social public services by the ecological immigrants and the indigenous people is shown in Fig. 6. We found a difference in the frequency of medical treatment requests by ecological migrants and indigenous people, with the indigenous people being less inclined to visit medical centers. The frequencies of going to the bank and the supermarket were relatively similar between the ecological immigrants and the indigenous villagers. Large proportions of both reported going to the supermarket every week; however, the proportion of ecological immigrants not going to the bank was higher. Overall, we found little difference in the use of public social services between ecological migrants and indigenous people. The ecological immigrants appeared to be slightly less financially secure than the indigenous people, but they had more relatives and friends.

### 3.3 Representational space of ecological migrant villages: reconstruction of spiritual space

As an important part of migrants' lives, the spiritual space of ecological migrant villages carries migrants' expectations of their own cultural identity and prospects for happiness in the construction process. It is not only the place where migrants construct their own spiritual world but also reflects their change and adaptation to cultural customs.

**Fig. 7:**
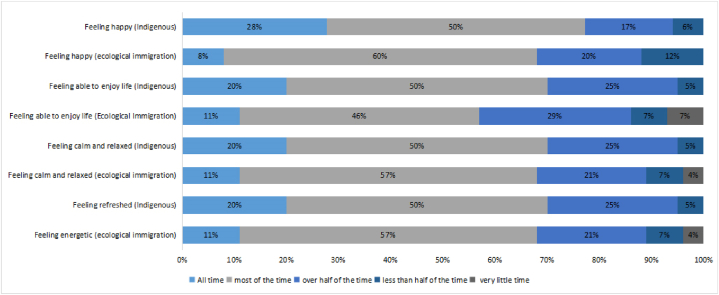
Wellbeing of ecological migrants and indigenous peoples.

**Fig. 8:**
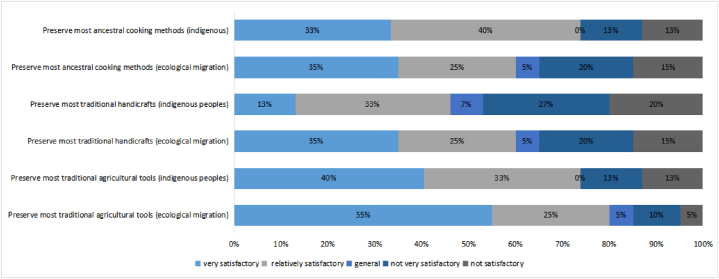
Ecological migration and preservation of indigenous traditions.

We compared the feeling of happiness and the preservation of traditions for the indigenous people and the ecological immigrants in Jiaochang Village, as shown in Fig. 7, Fig. 8. The indigenous people generally appeared to be slightly happier than the ecological migrants. Considering the four features of the spiritual space, i.e., feeling spiritual, feeling calm and relaxed, feeling able to enjoy life, and feeling happiness, the proportion of indigenous people reporting satisfaction was greater than that of ecological immigrants. Twenty percent of the indigenous people felt spiritual at all times, while only eleven percent of the ecomigrants did so. Twenty-eight percent of the indigenous people reported feeling happy at all times, while only eight percent of the ecomigrants did so. In general, ecological migrants may have felt less happy because they did not have a strong sense of belonging in their current residence and because of their lower living standards and finances in comparison with those of the indigenous people.

We found some differences between the ecological immigrants and the indigenous villagers in the preservation of traditional farm tools, traditional handicrafts, and ancestral cooking methods. The ecological migrants appeared more prone to preserving their traditional farming tools, traditional handicrafts, and ancestral cooking methods, while the indigenous people tended to preserve only their traditional cooking methods. This may suggest that when ecological migrants move to a new place, they try to retain their traditions. In this case, we propose that the ecological migrants, who originally lived in remote mountains, maintained their ancient habits after moving because they were not used to adopting new ones.

### 3.4 Comprehensive comparison

After the data obtained from the analysis of the three hierarchical spaces were normalized, we reported them in Table 2. Then, a *t*-test was carried out, as shown in Table 3. According to the results, the following conclusions can be drawn. We found significant differences between the mean values for the residential space, social space, and spiritual space and the reference value of 0. Both the *t*-value and the Sig. (two-tailed) indicated that the differences were statistically significant. Therefore, the living space, social space, and spiritual space of the ecological migrants and indigenous peoples were different. The difference in the spiritual space was the greatest, whereas that in social space was the least.

**Table 2:** Comparison of the results after data normalization.

Level	Eategory	Ecological Migrants	Indigenous People of Jiaochang Village
Living space	Homestead area	0.1823	0.2719
	Floor area	0.3764	0.4932
	Courtyard area	0.0545	0.2107
	Number of rooms	0.2375	0.2958
	Number of bedrooms	0.3	0.4061
	Number of toilets	0.1192	0.1795
	Homestead size satisfaction	0.3919	0.5446
	Housing satisfaction	0.6776	0.6607
	Traffic satisfaction	0.7297	0.7273
	Environmental satisfaction	0.7121	0.7222
Social space	wage	0.16	0.2535
	Household savings	0.0479	0.1329
	Unemployment rate	0.2	0.1379
	Number of Spring Festival visitors	0.398	0.3639
	Number of visits to the medical center	0.4012	0.3214
	Bank account		
	Bank visits	0.3681	0.354
	Trips to the supermarket	0.6889	0.7016
Living space	Happy and energetic	0.6607	0.7125
	Calm and relax	0.6607	0.7125
	Enjoying life	0.6161	0.7125
	Feel happy	0.66	0.75
	Keep most of the traditional farm tools	0.7875	0.6833
	Most of the traditional handicrafts are preserved	0.6125	0.4833
	Much of the ancestral cooking has been preserved	0.6125	0.6667

**Table 3:** Comprehensive comparison.

	t	Degree of Freedom	Sig. (Two-Tailed)	Mean Difference	Lower Bound of Confidence Interval	Upper Limit of Confidence Interval
Living space	4.296	9	0.002	0.07695	0.0364	0.1175
Social space	4.206	6	0.006	0.05432	0.0227	0.0859
Living space	7.153	6	0	0.0825	0.0543	0.1107

## 4 Discussion

By focusing on Jiaochang village, a typical ecological migrant village in Sichuan Province, this paper analyses the production process and characteristics of the new space of the ecological migrant village from three aspects, namely, spatial practice, spatial representation and representational space, using the analytical framework of tripartite dialectics in Lefebvre's spatial production theory. In the production of ecological migrant villages, spatial practices are mainly manifested as the reconstruction of living space, that is, the change in geographic location and spatial size of the homestead. There are certain differences between ecological migrants and indigenous locals in the acquisition and use of homesteads. The representation of space in the ecological migrant village is the reconstruction of social space; that is, the local government conceptualizes the relocation task of migrants and provides necessary social public services and support through the reconstruction of social space to maintain social order and stability. The space represented in the construction of ecological migrant villages is mainly manifested as the reconstruction of spiritual space, that is, the change in the feeling of happiness and inherent cultural customs of migrants before and after relocation. Specific conclusions are as follows:

This study found that, in terms of living space, indigenous people reported larger homestead areas, building areas, courtyard areas, numbers of rooms, numbers of bedrooms, and numbers of toilets than ecological immigrants did. This indicates that indigenous people may enjoy more living space and resources, though the housing satisfaction of the two groups is similar. Two possible reasons may explain this observation. First, when the ecological migrants moved to the new village, they could settle only on small plots, as most space was already occupied by the indigenous villagers. Therefore, their houses were small, and their satisfaction with their homesteads appeared to be slightly worse than that of the indigenous villagers. Second, the conditions of the original houses of the ecological immigrants were not good, and the houses were old; so, the migrants' satisfaction with the new housing after relocation was found to be high. In addition, the original houses had few bedrooms and no bathrooms, while the numbers of bedrooms and bathrooms in the new houses after the relocation were only slightly lower than those in the indigenous people's houses.

In terms of social space, the indigenous people reported higher wages and household savings than the ecological migrants did. The ecomigrants appeared to have similar job networks to those of the indigenous people, though the unemployment rates were slightly higher among the ecomigrants than among the indigenous people, who appeared to have relatively stable employment. The ecomigrants reported visiting slightly more people than the indigenous villagers during the Spring Festival and having more friends and relatives to visit. This may indicate that, even after the relocation, the ecological immigrants tended to maintain relatively close contact with their relatives and friends from the same village and ethnic group, as they still lived together. At the same time, they became gradually integrated into the community to which they were relocated. Sundstrom argues that social spaces are difficult to integrate while maintaining ethnic and racial identity [45]. The composition of the indigenous ethnic group in this study was complex, comprising Han, Tibetan, and Qiang villagers with relatives and friends who did not live close by, so the number of people visiting relatives and friends was smaller for this group.

Before the relocation, the ecological migrants were mainly farmers, but after the relocation, most of them chose other ways to make a living, feeling too far from their own farming land. Their job network appeared close to that of the indigenous villagers, but their financial situation was not as good and will require some time to improve.

In terms of spiritual space, compared with the indigenous people, the ecological immigrants appeared to be less happy, spiritual, calm, and relaxed and to be less able to enjoy life. Regarding the preservation of traditions, the ecological migrants appeared to retain their traditional farming tools, traditional handicrafts, and ancestral cooking methods. Overall, considering the differences that we found for the three levels of space, the difference for the social space was the lowest, while that for the spiritual space was the highest.

The ecological migration policy after the Wenchuan earthquake demonstrates the act of moving a population from its original residence to other areas due to the deterioration of the original ecological environment and the unsuitability of the original village sites for survival with the goal of providing better living conditions for the relocated residents. Thirteen years have passed since the time of this survey. During these 13 years, the multi-level space of ecological migration has undergone a reconstruction. According to the current investigation results, the reconstruction of the living space and social space of the eco-immigrants in Jiaochang Village has basically been completed. The residents are basically satisfied with their housing conditions, although they are very different from those in their original home. The use of public social services by the residents is also very good. However, with respect to jobs and finances, the ecomigrants’ situation needs improvement. There are still differences in the spiritual space for the migrants and the indigenous people in Jiaochang Village, and it will take some time to reconstruct a satisfying spiritual space for the ecomigrants.

We performed an in-depth study of spatial reconstruction for ecomigrants in Jiaochang Village. The main innovations of this research are the study's method and structure. In the past, few researchers conducted in-depth investigations on villages to study the specific situations of ecological migrants. In previous studies, the research on ecological migration in China primarily focused on the environmental impacts of ecological migrants [46,47]. Some studies also show results similar to ours. For example, research on the ecological migrants in the Sanjiangyuan wetlands indicates that the main issue they face is job maladaptation [48]. In the Ningxia region, the care for elderly ecological migrants is not adequately secured, highlighting issues at the social spatial level [49]. Additionally, studies on the spiritual space have shown that the subjective well-being of poverty alleviation migrants in Guizhou needs improvement. Our results showed some differences in satisfaction between ecological immigrants and indigenous people, but it was difficult to identify the most critical aspects that need improvement. The results of this research show that ecological immigrants can gradually integrate into multi-ethnic villages while retaining their own characteristics; however, the spiritual space was still unsatisfactory and needs special attention.

## Data availability statement

The data from this study are not stored in a publicly accessible repository due to concerns over the privacy of the participants involved.

## Review reports

The research program and procedures complies with national laws and the rules and regulations of BJFU, and all are handled in accordance with the requirements of the **Human Study Ethics Committee** of BJFU. The proposal has been reviewed and approved by the Human Research Ethics Committee of BJFU.

Approval No. BJFUPSY-2023-017.

## Informed consent statement

### Participant consent for research study

I, Xueyue Bai, the undersigned, hereby confirm that I have conducted a research study on ecological migration. The study involved the collection of survey responses from participants who willingly and voluntarily agreed to participate. This informed consent statement is intended to affirm that all participants in this study were informed of the study's purpose and procedures and provided their consent to participate.

I, Xueyue Bai, the principal investigator, and researcher for this study, affirm the following:

Purpose: The purpose of this research study was to investigate the living conditions and experiences of individuals involved in ecological migration.

Procedures: Participants were invited to complete a questionnaire or survey related to their experiences with ecological migration. The survey included questions about their living conditions, challenges faced, and overall well-being.

Confidentiality: All information collected from participants during this study was kept confidential and anonymous. Participants were assured that their identities and individual responses would not be disclosed or shared with any third parties.

Voluntary Participation: Participants were informed that their participation in this study was entirely voluntary. They were under no obligation to participate, and they could withdraw from the study at any time without any consequences.

Awareness: All participants were made aware of the study's purpose, procedures, and the intended use of the data collected.

I confirm that I have taken all necessary measures to obtain informed consent from each participant in this research study. Participants were given clear information about the study and provided their consent willingly.

This informed consent statement serves as documentation that all participants in this study were informed and agreed to participate voluntarily, with full awareness of the study's purpose and the use of their responses for research on ecological migration.

## CRediT authorship contribution statement

**Xueyue Bai:** Writing – review & editing, Writing – original draft, Project administration, Methodology, Data curation, Conceptualization. **Qihui Liu:** Writing – original draft, Methodology, Data curation. **Jingtian Ge:** Writing – original draft. **Cong Dong:** Writing – review & editing.

## Declaration of competing interest

The authors declare that they have no known competing financial interests or personal relationships that could have appeared to influence the work reported in this paper.
